# Supplementary material for: Subsurface A-site vacancy activates lattice oxygen in perovskite ferrites for methane anaerobic oxidation to syngas
Source: Nat Commun. 2024 Jun 26;15:5422. doi: 10.1038/s41467-024-49776-y (PMC11208437; doi:10.1038/s41467-024-49776-y)
Supplement: Supplementary file 1 — Supplementary Information [file 41467_2024_49776_MOESM1_ESM.pdf]

Supplementary information

Subsurface A-site vacancy activates lattice oxygen in perovskite ferrites for methane anaerobic oxidation to syngas

Jiahui He[1,2,†], Tengjiao Wang[3,†], Xueqian Bi[1,4], Yubo Tian[1,5], Chuande Huang[1,], Weibin Xu[1,6], Yue Hu[1,6], Zhen Wang[1,6], Bo Jiang[3,*], Yuming Gao[3], Yanyan Zhu[2,*], Xiaodong Wang[1,*]*

[1]CAS Key Laboratory of Science and Technology on Applied Catalysis, Dalian Institute of Chemical Physics, Chinese Academy of Sciences, Dalian 116023, China

[2]School of Chemical Engineering, Northwest University, International Scientific and Technological Cooperation Base of MOST for Clean Utilization of Hydrocarbon Resources, Chemical Engineering Research Center for the Ministry of Education for Advance Use Technology of Shanbei Energy, Xi'an 710069, China

[3]Key Laboratory of Ocean Energy Utilization and Energy Conservation of Ministry of Education, Dalian University of Technology, Dalian 116023, China

[4]College of Environmental Science and Engineering, Dalian Maritime University, Dalian 116026, China

[5]School of Chemical Engineering, Zhengzhou University, Zhengzhou 450001, P. R. China

[6]School of Chemical Engineering, University of Chinese Academy of Science, Beijing 100049, China

[†]*These authors contributed to this work equally.*

*Corresponding author

E-mail address:

huangchuande@dicp.ac.cn (C.D. Huang);

bjiang@dlut.edu.cn (B. Jiang);

zhuyanyan@nwu.edu.cn (Y.Y. Zhu);

xdwang@dicp.ac.cn (X.D. Wang);

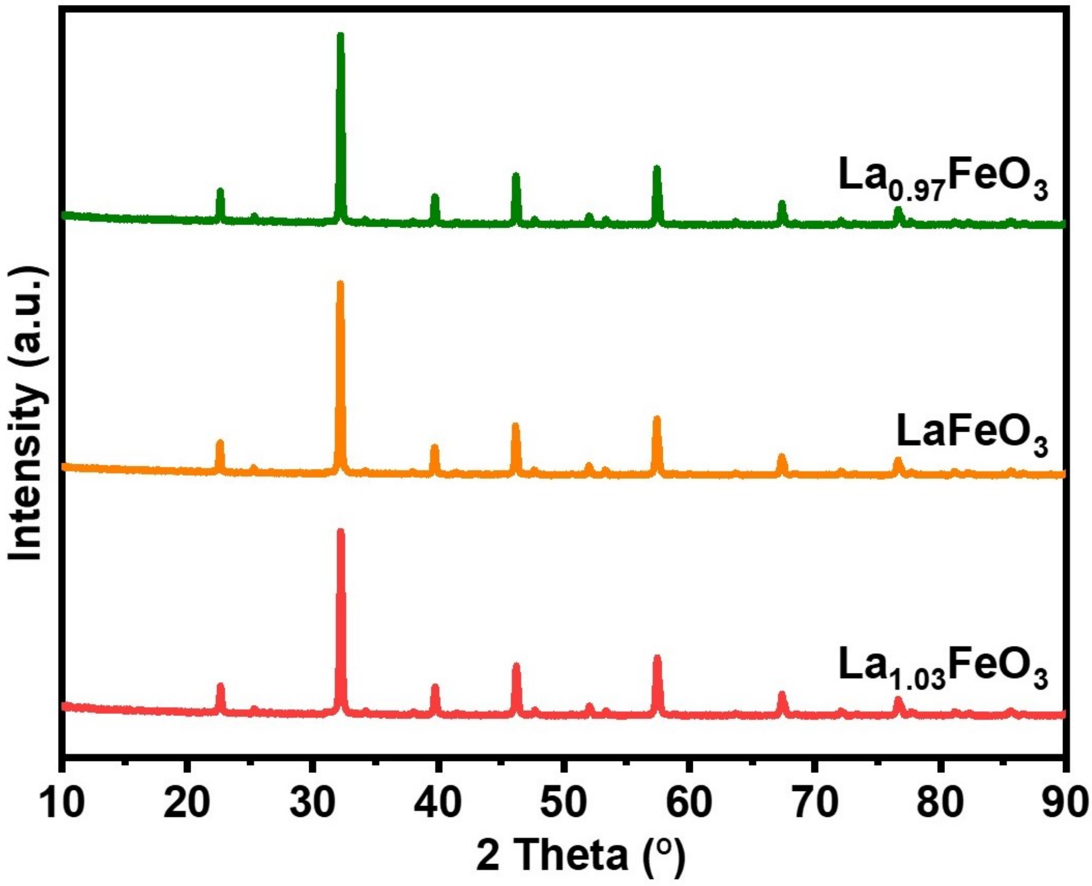

Figure S1. XRD patterns of La_xFeO_3 (x = 1.03, 1, and 0.97) oxides.

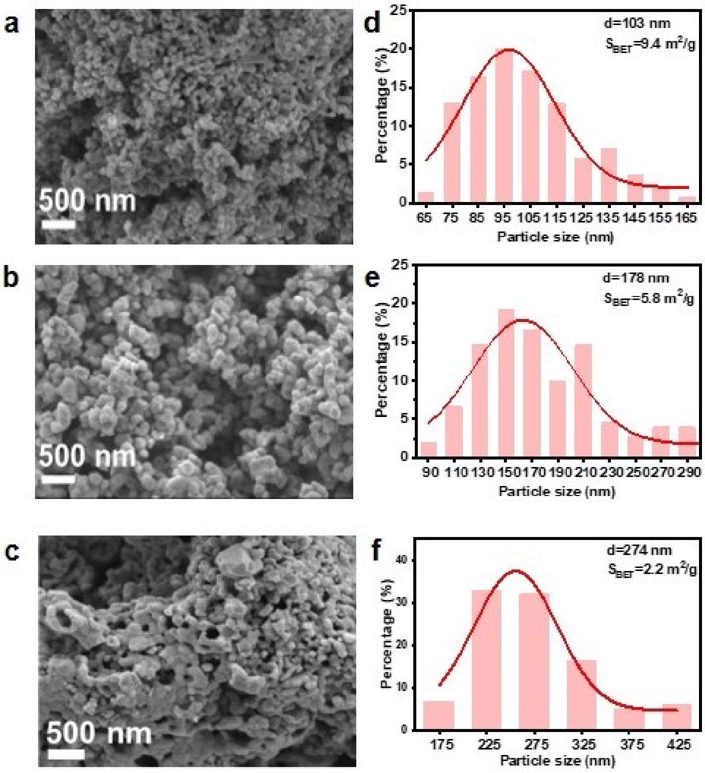

Figure S2. SEM images of fresh samples. SEM images of fresh **a** $La_{1.03}FeO_3$, **b** $LaFeO_3$, and **c** $La_{0.97}FeO_3$, and **d-f** the corresponding grain size statistical results.

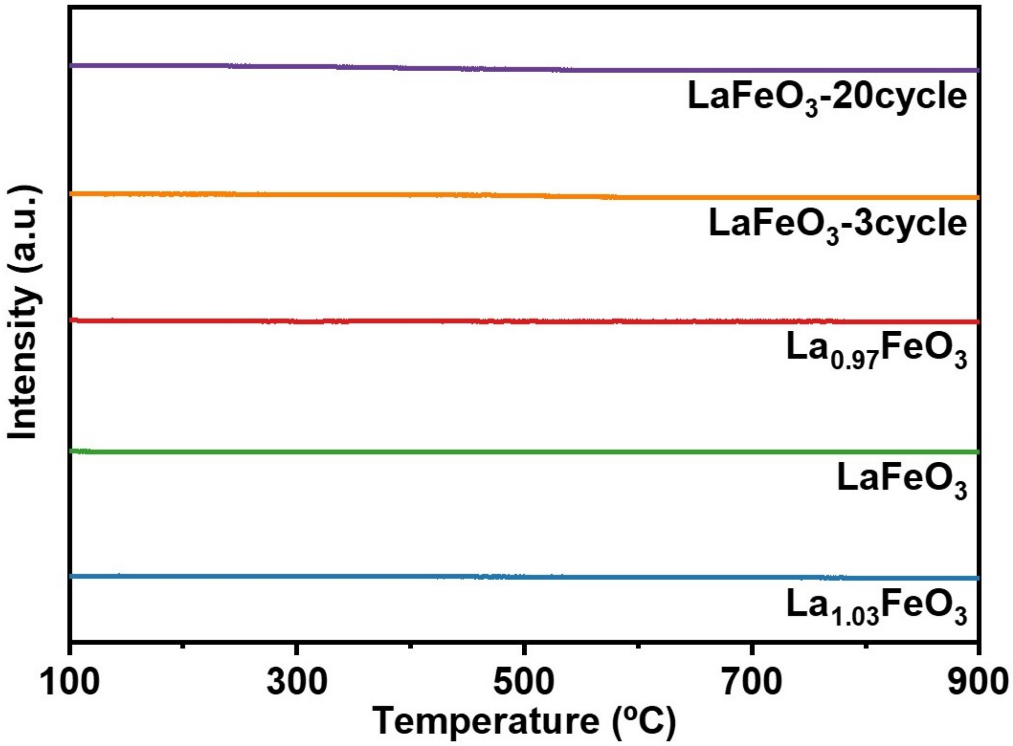

Figure S3. O_2**-TPD profiles for** La_xFeO_3 **(x = 1.03, 1, and 0.97) of fresh and cycled oxides.**

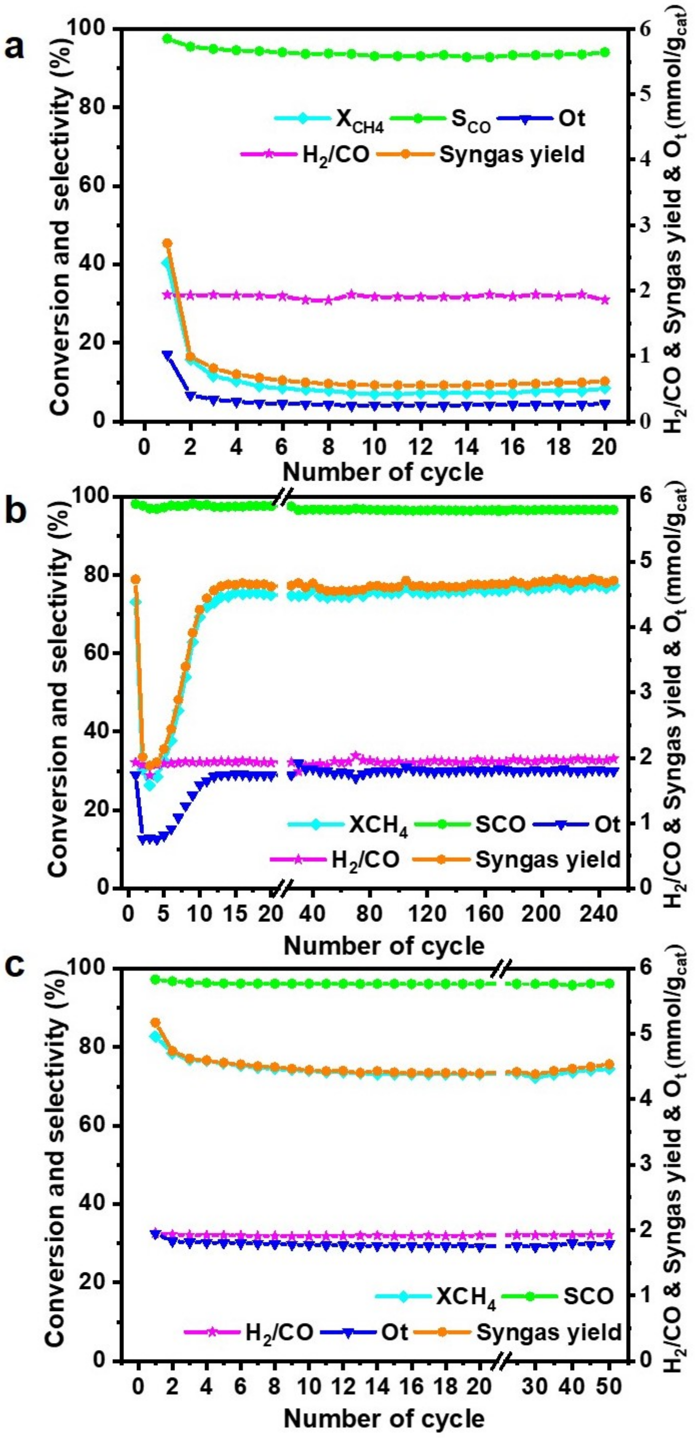

Figure S4. Stability tests of catalysts. The performance of CH_4 partial oxidation step for **a** $La_{1.03}FeO_3$ during 20 cycles, **b** $LaFeO_3$ during 250 cycles, and **c** $La_{0.97}FeO_3$ during 50 cycles. Reaction conditions: 100 mg catalyst treated with 5% CH_4/He (15 mL/min) for 8 min during CH_4 partial oxidation step, 5% CO_2/He (15 mL/min) for 10 min during CO_2 regeneration step at 900 °C, and the reactor was purged with He for 4 min (20 ml/min) between partial oxidation and reoxidation step.

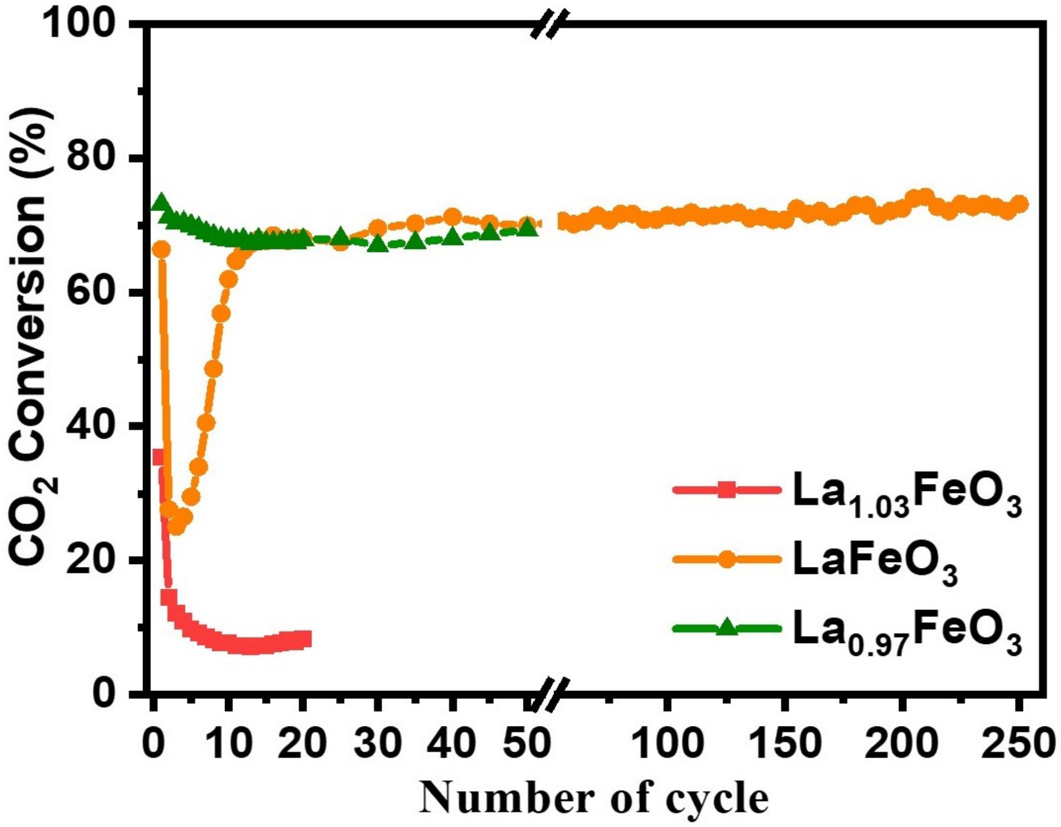

Figure S5. CO_2 conversion of regeneration step. Reaction conditions: 100 mg catalyst was treated with 5% CH_4/He (15 mL/min) for 8 min during CH_4 partial oxidation step, 5% CO_2/He (15 mL/min) for 10 min during CO_2 regeneration step at 900 °C, and the reactor was purged with He for 4 min (20 ml/min) between partial oxidation and reoxidation step.

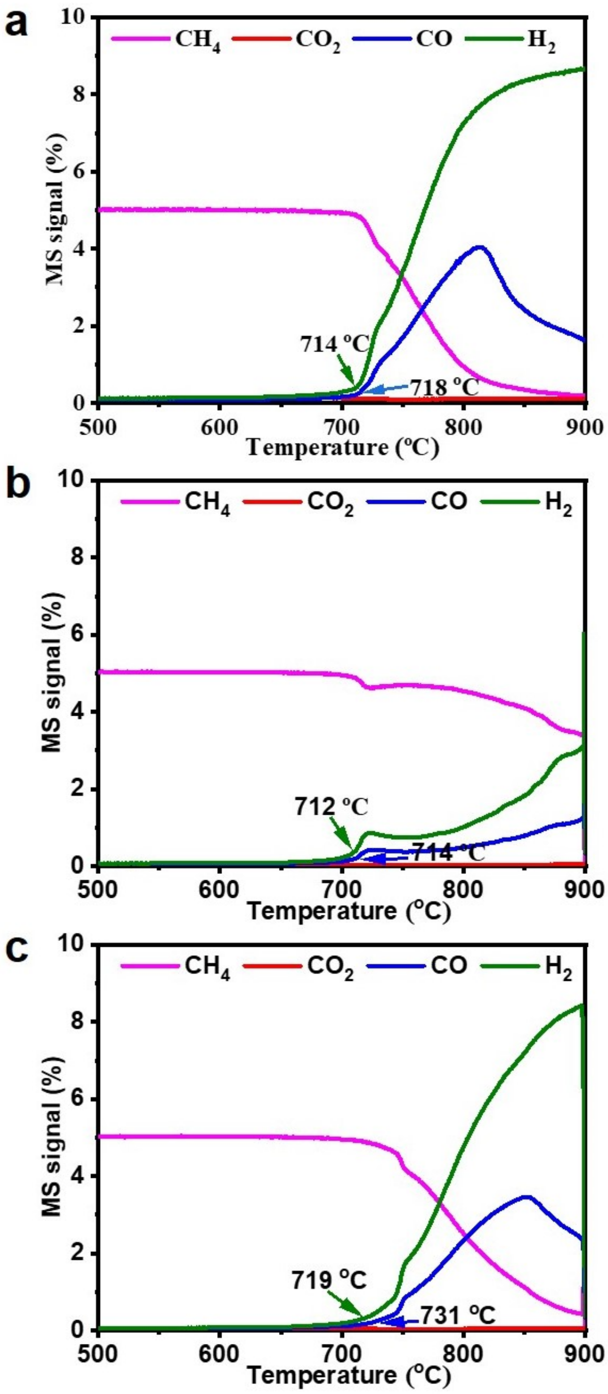

Figure S6. CH_4-TPR profiles. a $LaFeO_3$, **b** $LaFeO_3$-3, and **c** $LaFeO_3$-20. Reaction conditions: 100 mg catalyst treated with 5% CH_4/He (30 mL/min) from 20 °C to 900 °C with a ramp rate of 10 °C/min.

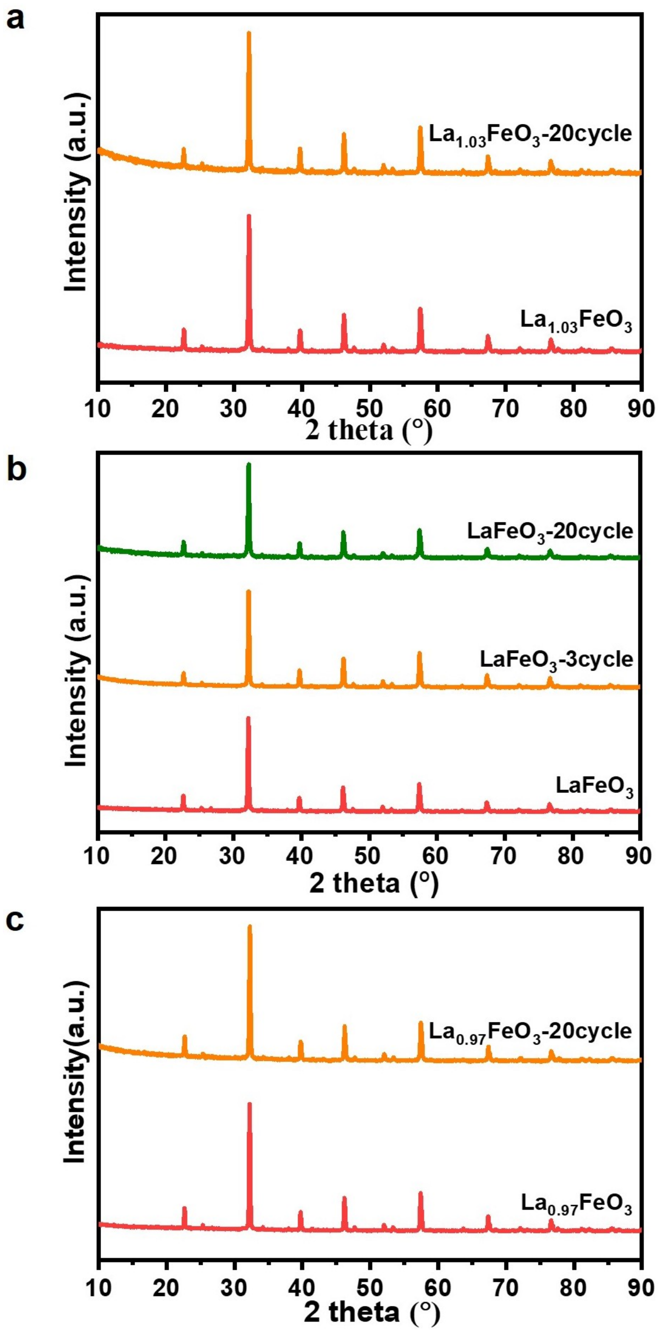

Figure S7. XRD patterns of fresh and cycled samples. a $La_{1.03}FeO_3$, **b** $LaFeO_3$, and **c** $La_{0.97}FeO_3$.

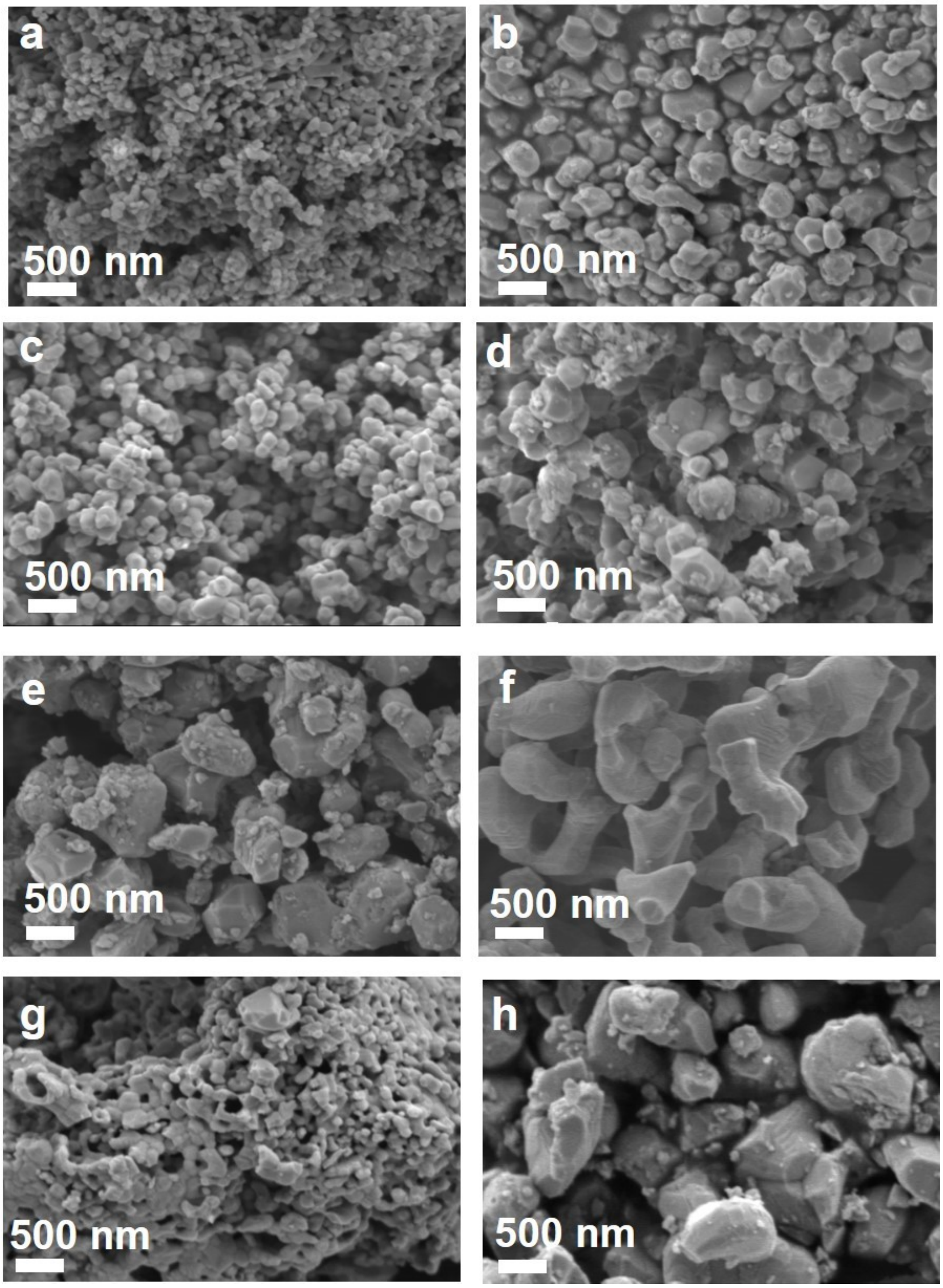

Figure S8. SEM images of fresh and cycled samples. **a** $La_{1.03}FeO_3$, **b** $La_{1.03}FeO_3$-20, **c** $LaFeO_3$, **d** LaFeO3-3, **e** $LaFeO_3$-20, **f** $LaFeO_3$-250, **g** $La_{0.97}FeO_3$, and **h** $La_{0.97}FeO_3$-20.

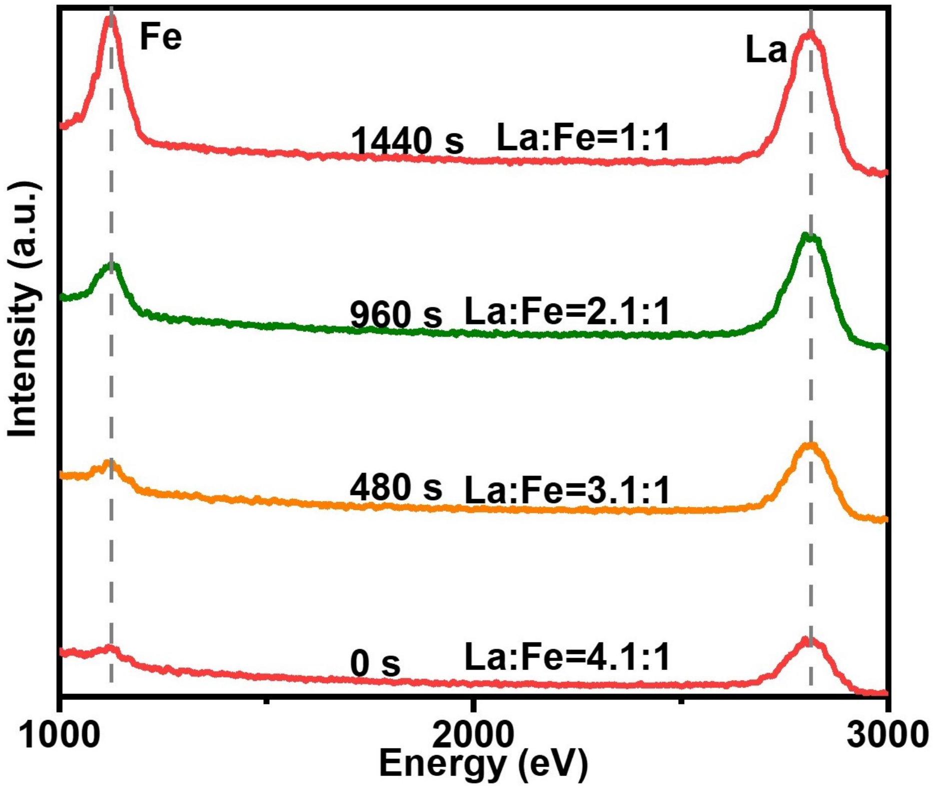

Figure S9. Variation in the LEIS peak signal of La and Fe atoms with sputtering time.

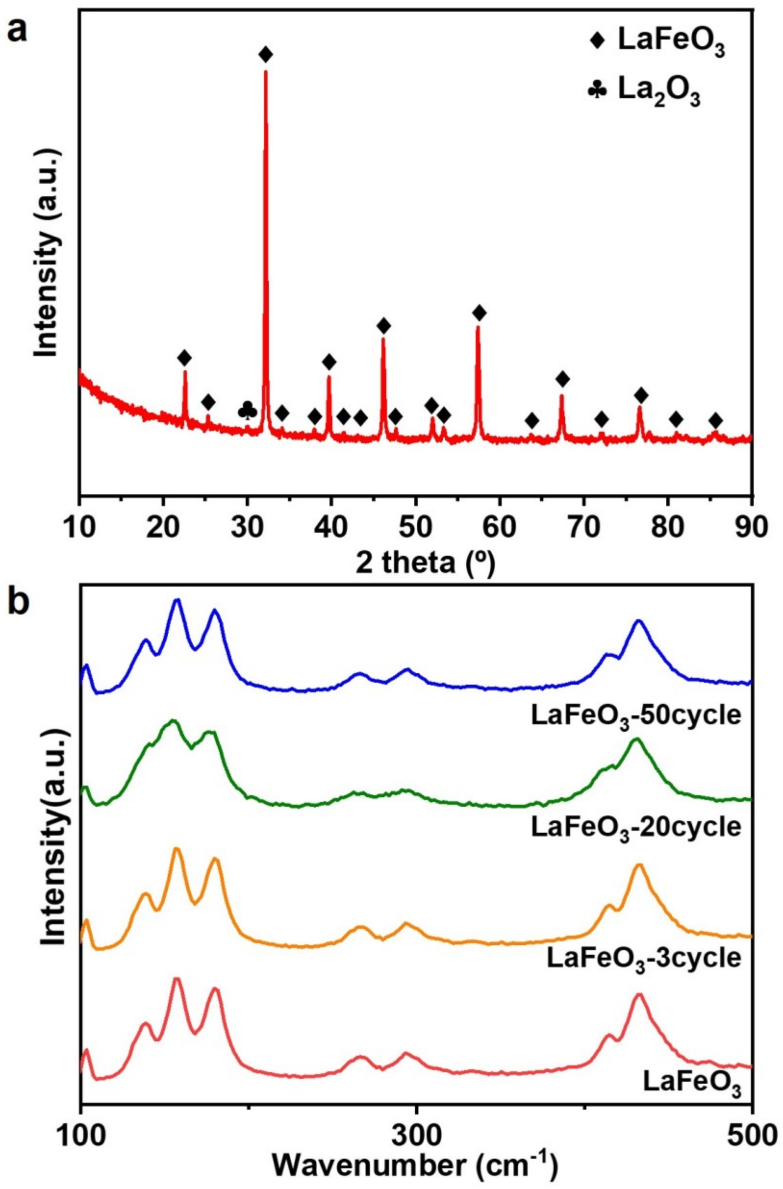

Figure S10. XRD and Raman spectra of the catalysts. a XRD pattern of $LaFeO_3$-50, and **b** Raman spectra of fresh and cycled $LaFeO_3$.

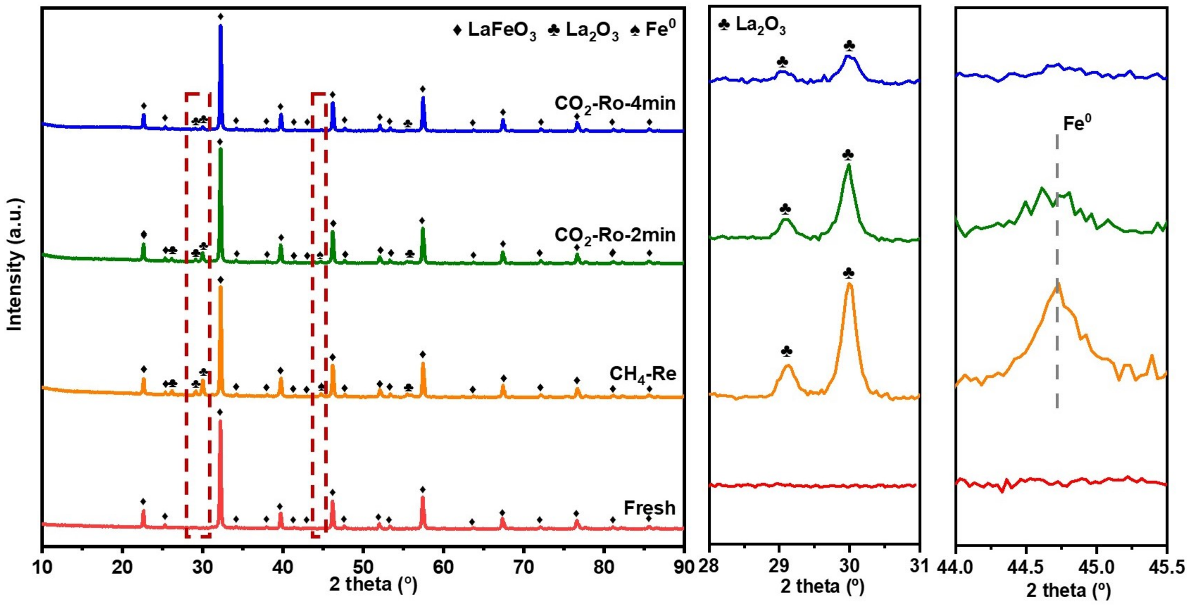

Figure S11. XRD patterns of fresh $LaFeO_3$, $LaFeO_3$ after CH_4 reduction and different regeneration time in CO_2 with corresponding magnified view in the range of 28-31° and 44.0−45.5°. ($LaFeO_3$ was reduced by CH_4 and marked as CH_4-Re; the reduced $LaFeO_3$ was oxidized by CO_2 for 2 min and recorded as CO_2-Ro-2min; and the reduced $LaFeO_3$ was oxidized by CO_2 for 4 min and recorded as CO_2-Ro-4min).

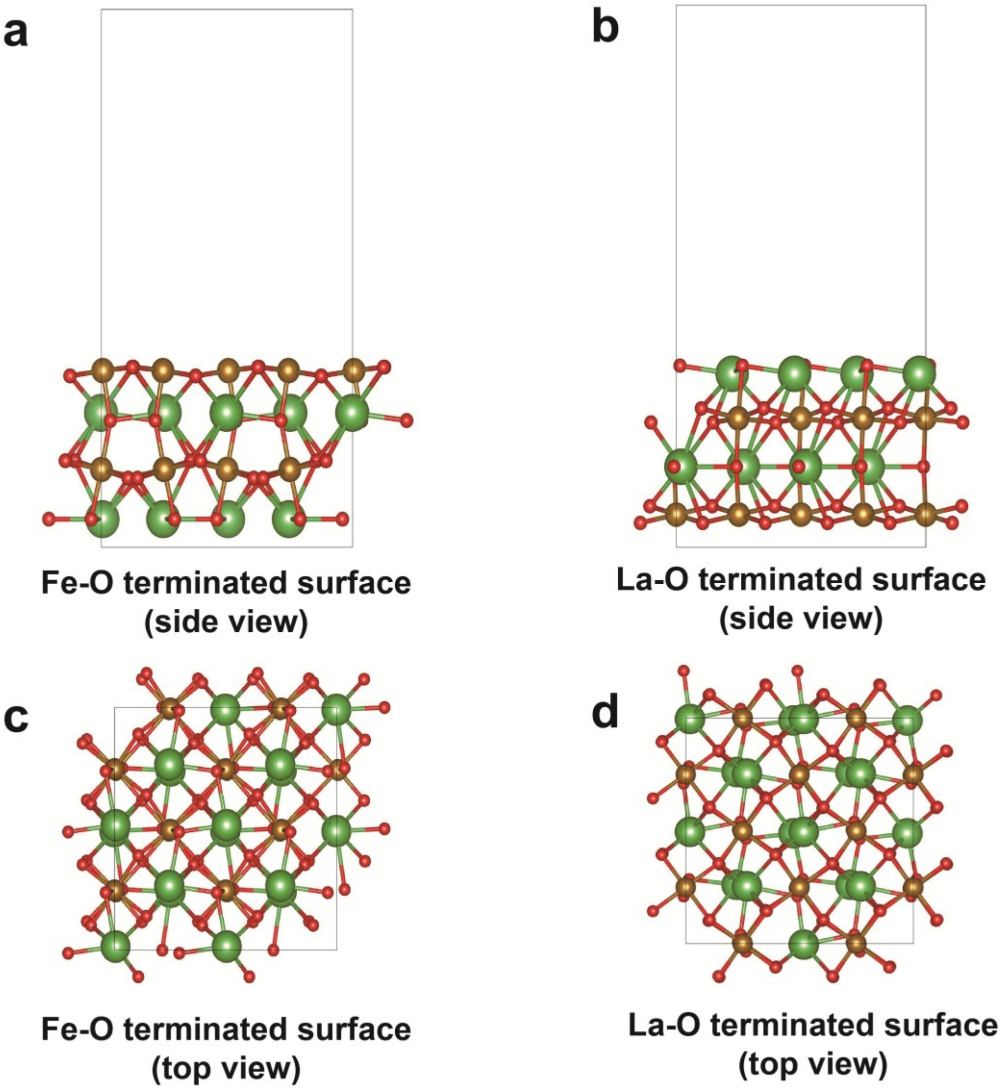

Figure S12. Surface structures for $LaFeO_3$. Fe-O terminated surface for $LaFeO_3$ through **a** side view and **c** top view, and La-O terminated surface for $LaFeO_3$ through **b** side view and **d** top view.

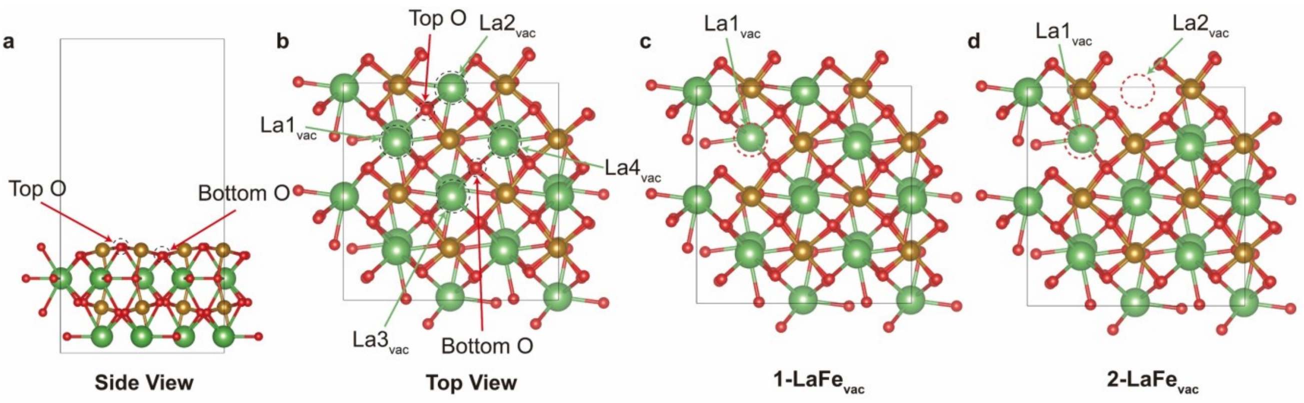

Figure S13. Fe-O terminated surface for $LaFeO_3$. Fe-O terminated surface for $LaFeO_3$ through **a** side view and **b** top view, and **c** $LaFeO_3$ with single La vacancy, and **d** $LaFeO_3$ with double La vacancies.

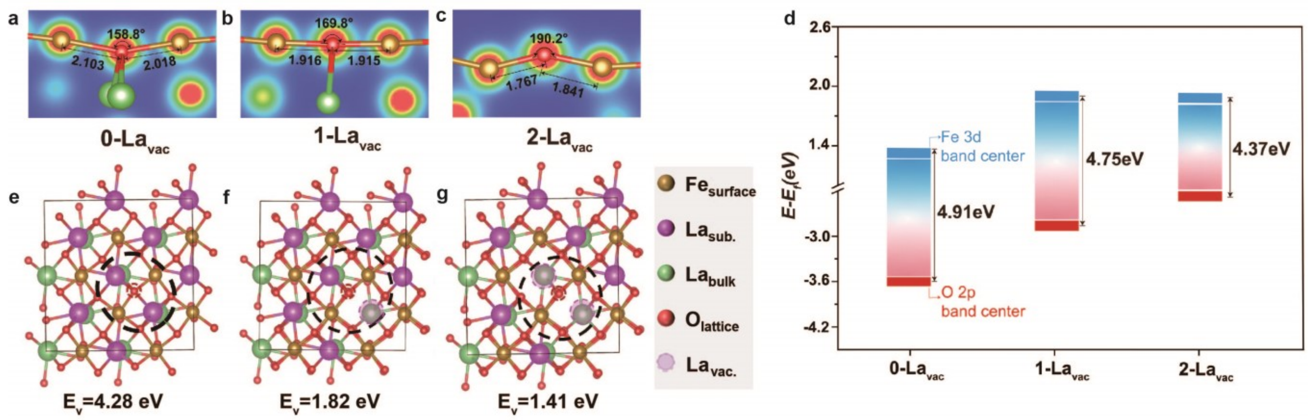

Figure S14. Theoretical investigations on the effect of subsurface La vacancies. a-c surface structure changes with $La_{sub.}$ vacancy concentration for the bottom O, **d** Charge-transfer energy with corresponding band centers of unoccupied Fe *3d* and occupied O *2p* states for different $La_{sub.}$ vacancy concentration for the bottom O, and **e-g** Computational model of oxygen vacancy formation in bulk of different $La_{sub.}$ vacancy concentration based on the bottom O. ($La_{vac.}$ stands for subsurface La vacancy; $La_{sub.}$ stands for subsurface La).

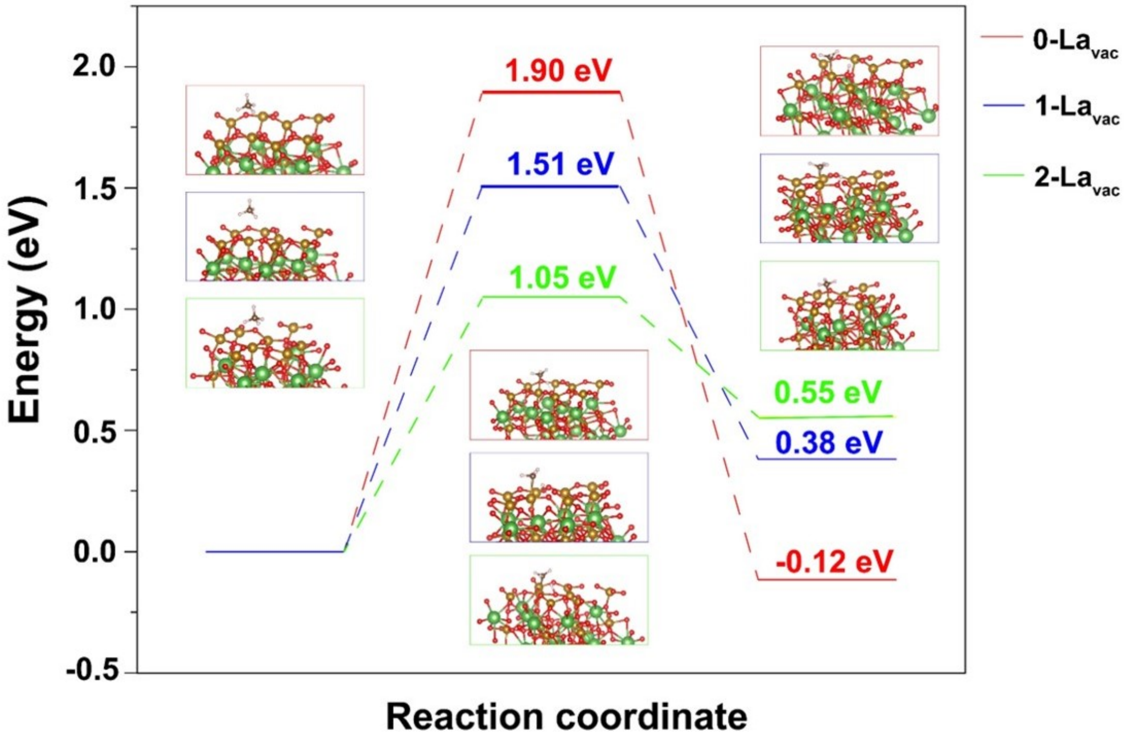

Figure S15. Comparison of energy profile of CH_4 activation (TS1) over bottom oxygen coordinated with different number of $La_{sub.}$ vacancies. $La_{vac.}$ stands for subsurface La vacancy.

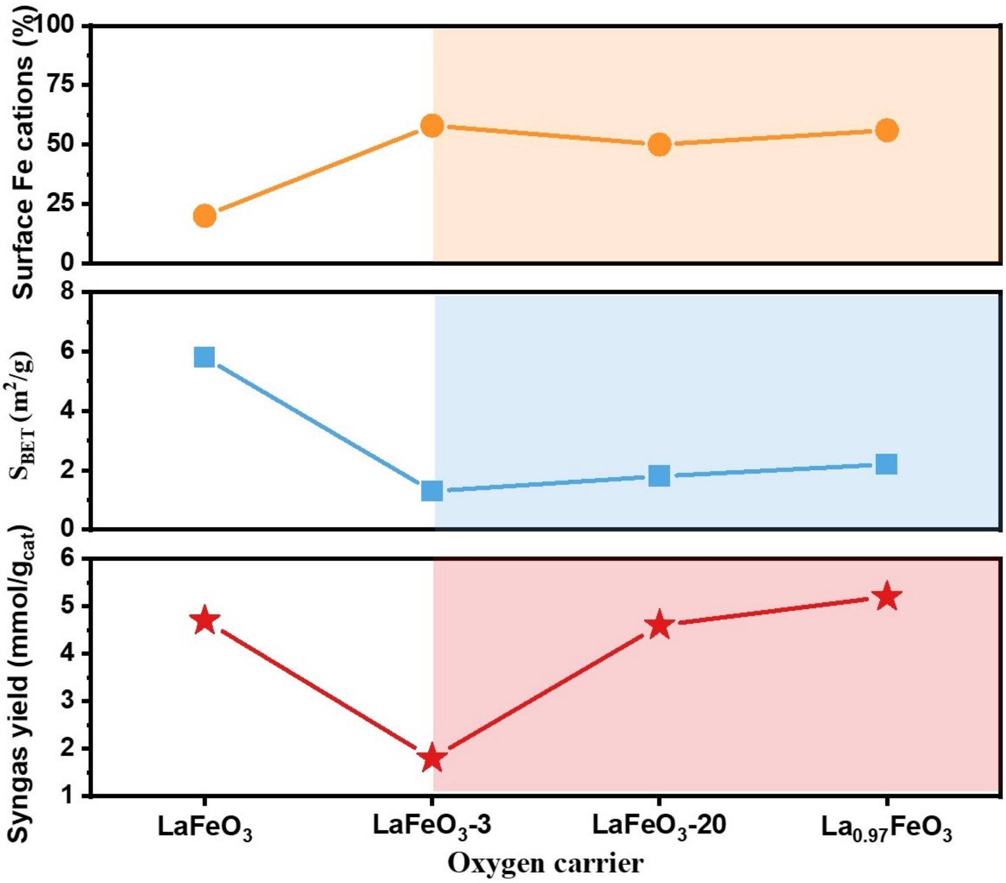

Figure S16. Correlation between specific surface area, surface Fe percentage and the corresponding chemical looping methane conversion performance.

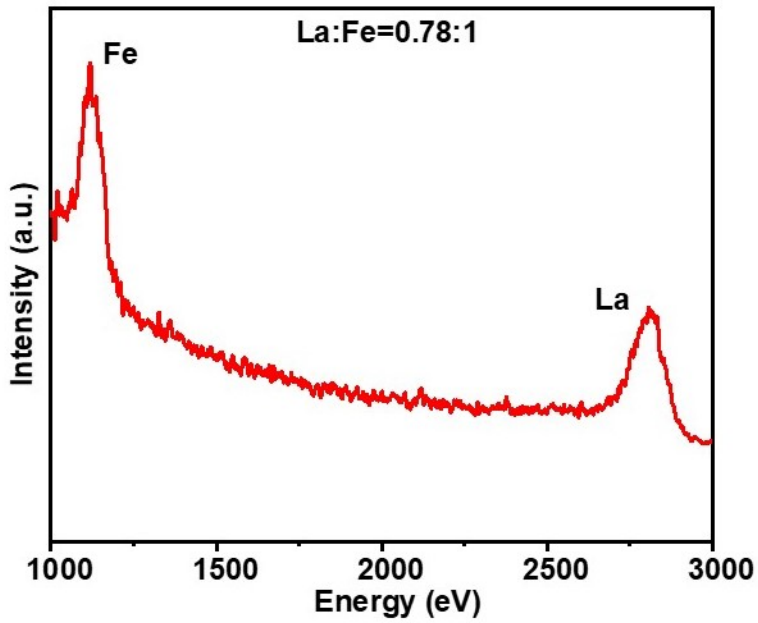

Figure S17. The LEIS peak signal for La and Fe atoms at the first layer of fresh $La_{0.97}FeO_3$ oxides.

Table S1. Quantitative results of XPS for La_xFeO_3 (x = 1.03, 1, and 0.97) of fresh and cycled oxides.

Sample	La/Fe[a]	O^{2-} (%) [b]	O_2^{2-}/O^- (%) [c]	CO_3^{2-}/OH^- (%) [d]	H_2O (%) [e]
$La_{1.03}FeO_3$	2.41	34.45	12.30	36.75	16.50
$LaFeO_3$	1.22	35.07	14.86	34.77	15.30
$La_{0.97}FeO_3$	0.91	46.55	17.78	24.64	11.03
$La_{1.03}FeO_3$-20	2.10	37.93	11.43	38.83	11.81
$LaFeO_3$-20	1.58	35.41	12.20	40.28	12.11
$La_{0.97}FeO_3$-20	1.49	36.70	12.01	39.33	11.96

[a] La/Fe ratio.

[b] Percentage of O^{2-} in oxygen species.

[c] Percentage of O_2^{2-}/O^- in oxygen species.

[d] Percentage of CO_3^{2-}/OH^- in oxygen species.

[e] Percentage of H_2O in oxygen species.

Table S2. Total energy for different terminated surfaces.

Different terminated surface	Total energy (eV)
Fe-O terminated surface	-598.59
La-O terminated surface	-599.33

Table S3. Total energy for different La vacancies.

Different La vacancies	Total energy (eV)
$La1_{vac}$	-583.15
$La3_{vac}$	-583.14
$La1_{vac}$ and $La2_{vac}$	-567.09
$La3_{vac}$ and $La4_{vac}$	-567.13

Table S4. Oxygen vacancy formation energy for different La vacancies.

Oxygen vacancy formation energy (eV)	Top O	Bottom O
0-$LaFe_{vac}$	3.25	4.28
1-$LaFe_{vac}$	1.37	1.82
2-$LaFe_{vac}$	0.79	1.41

Table S5. Basic parameters for different La_{sub} vacancy concentrations from DFT calculations for the bottom O.

Subsurface La vacancy number	0-$La_{vac.}$	1-$La_{vac.}$	2-$La_{vac.}$
Charge transfer energy	4.91 eV	4.75 eV	4.37 eV
Bader charge (Fe)	1.67	1.68	1.69
Bader charge (O)	-1.22	-1.19	-1.15
Oxygen vacancy formation energy	4.28 eV	1.82 eV	1.42 eV
Hydrogen atom adsorption energy	-1.39 eV	-1.77 eV	-1.95 eV

Table S6. Basic parameters for different La_{sub} vacancy concentrations from DFT calculations for the top O.

Subsurface La vacancy number	0-$La_{vac.}$	1-$La_{vac.}$	2-$La_{vac.}$
Charge transfer energy	4.87 eV	4.69 eV	4.31 eV
Bader charge (Fe)	1.64	1.66	1.67
Bader charge (O)	-1.11	-1.07	-1.02
Oxygen vacancy formation energy	3.25 eV	1.37 eV	0.79 eV
Hydrogen atom adsorption energy	-1.43 eV	-1.88 eV	-2.04 eV

Table S7. Energy barrier for methane partial oxidation reaction.

Energy barrier (E_v)	0-$La_{vac.}$	1-$La_{vac.}$	2-$La_{vac.}$
TS1	1.88	1.46	1.03
TS2	1.84	1.39	0.99
TS3	1.67	1.22	0.77
TS4	1.82	1.23	0.75

Table S8. The BET specific surface area of La_xFeO_3 (x = 1.03, 1, 0.97) and cycled $LaFeO_3$ catalyst.

Sample	$La_{1.03}FeO_3$	$LaFeO_3$	$La_{0.97}FeO_3$	$LaFeO_3$-3	$LaFeO_3$-20
S_{BET} (m^2/g_{cat})	9.4	5.8	2.2	1.3	1.8
